# Explainable AI: Ethical Frameworks, Bias, and the Necessity for Benchmarks

**DOI:** 10.1055/a-2702-1843

**Published:** 2025-09-23

**Authors:** Rosa Verhoeven, Wiam Bouisaghouane, Jan BF Hulscher

**Affiliations:** 1 Department of Surgery, Division of Pediatric Surgery, University Medical Center Groningen, University of Groningen, The Netherlands; 2 Division of Neonatology, Beatrix Children's Hospital, University Medical Center Groningen, University of Groningen, Groningen, The Netherlands

**Keywords:** explainable AI, machine learning, bias, pediatric surgery, benchmarks

## Abstract

Artificial intelligence (AI) is increasingly integrated into pediatric healthcare, offering opportunities to improve diagnostic accuracy and clinical decision-making. However, the complexity and opacity of many AI models raise concerns about trust, transparency, and safety, especially in vulnerable pediatric populations. Explainable AI (XAI) aims to make AI-driven decisions more interpretable and accountable. This review outlines the role of XAI in pediatric surgery, emphasizing challenges related to bias, the importance of ethical frameworks, and the need for standardized benchmarks. Addressing these aspects is essential to developing fair, safe, and effective AI applications for children. Finally, we provide recommendations for future research and implementation to guide the development of robust and ethically sound XAI solutions.

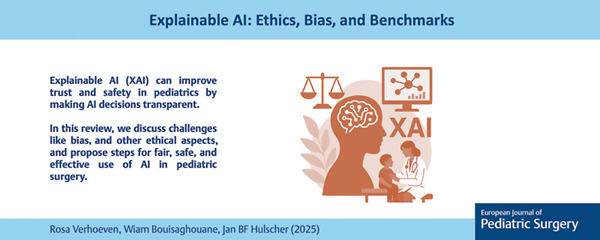

## Introduction

Artificial intelligence (AI) refers to the capability of machines and software systems to perform tasks that were typically thought to require human intelligence. At its core, AI leverages large datasets and complex algorithms to autonomously identify patterns and make classifications or predictions. In recent years, the availability of high-quality clinical data, advances in computing power, and the evolution of open-source algorithms have accelerated the adoption of AI across various domains of medicine. 1

One of the domains in which we have seen a rapid expansion of AI research is pediatrics. 2 In particular, it is increasingly used to support diagnostic accuracy and enhance prognostic assessments. 3 4 For instance, computer vision models have been developed to improve the diagnostic value of medical images in pediatric oncology, cardiology, and surgery. 5 6 7 8 Predictive models are being developed to anticipate complications such as acute appendicitis, sepsis and/or necrotizing enterocolitis, or postoperative transplant outcomes in pediatric patients. 9 10 11 12 13

However, pediatric populations present unique clinical and ethical challenges for AI deployment. Compared to adult datasets, pediatric data are often smaller, more heterogeneous, and dynamically evolving due to developmental stages. 3 14 This makes model training and validation more complex, increasing the risk of overfitting and limiting generalizability across different pediatric subgroups. Such challenges can result in biased algorithmic decisions, which are especially concerning for these vulnerable populations, as they may exacerbate existing health disparities or lead to inappropriate interventions with potential long-term effects on a child's development. Hence, explainable AI (XAI), which seeks to make algorithmic processes more transparent, interpretable, and comprehensible to human stakeholders, has become essential in pediatric contexts. 15 16 Unlike so-called “black-box” models, whose reasoning remains opaque, XAI systems aim to illustrate “how” and “why” specific predictions are made, often quantifying aspects such as feature importance, model limitations, and decision dependencies.

The objective of this review is to provide an overview of the current and potential role of XAI in pediatric surgery, with a focus on the challenges that are posed by bias, the ethical frameworks that guide its development, and the pressing need for standardized benchmarks that promote safe and efficient use of XAI.

## Bias in AI

Bias comes in many forms, and it can permeate every stage of the AI pipeline, from data collection and labeling to model development, evaluation, and interpretation of algorithmic decisions. Systematic reviews have shown that up to 77% of AI-based models in pediatrics have a high risk of bias, raising concerns about their clinical applicability and trustworthiness. 4 14

Bias often emerges as early as the data collection phase. 3 Many pediatric datasets are derived from research populations that are not fully representative of the broader pediatric community. This introduces selection or representation bias. For example, studies may exclude children with comorbidities or they may rely heavily on records from tertiary hospitals, resulting in samples that over-represent complex or severe cases and under-represent more routine pediatric cases. 17 Additionally, geographic and socioeconomic disparities might exacerbate representational inconsistencies. 18 A large portion of pediatric AI research is based on data from Western, high-income countries, which introduces systemic racial, ethnic, and socioeconomic bias. These datasets often fail to reflect the realities of children in low- and middle-income countries or marginalized communities. 19

Another layer of bias might emerge during the labeling process. Many AI models rely on supervised learning, which requires labeled data often annotated by clinicians. However, these clinical labels can be influenced by cognitive biases such as attribution bias (over-relying on one's own assumptions or experience) and availability bias (depending on easily recalled cases), especially in high-pressure environments. 20 21 22 These biases can propagate into the AI model during training, resulting in biased ground truth annotations that distort model learning.

During the model development phase, further biases may emerge. Algorithmic bias can result from the choice of modeling techniques, tuning of hyperparameters, or the metrics used to evaluate performance. 23 Developers may unconsciously favor models that confirm preexisting beliefs or expectations, which is a manifestation of confirmation bias. 24 25

Even after deployment, bias may persist or even amplify. Algorithms embedded in electronic health records or clinical decision support systems can reinforce existing inequities if they are not continuously monitored and recalibrated. A particularly important risk in this regard is automation bias, which is the tendency of clinicians to over-rely on algorithmic output, even when they may be flawed. 26 This potentially creates a feedback loop, where biased predictions influence clinical decisions, further skewing data used for future model retraining.

Some sources of bias, particularly those rooted in data collection, may be difficult or even impossible to eliminate entirely. Therefore, transparency, followed by an accurate understanding of how the AI model arrives at its predictions is essential, as it enables clinicians and researchers to detect potential biases in the model's reasoning process and take corrective measures where possible.

## XAI

Various model interpretability techniques can help clinicians understand what features the AI model is relying on, how confident it is in a given prediction, and whether its recommendations should be trusted or questioned in specific contexts. This helps to establish appropriate trust, preventing clinicians from over-relying on AI due to automation bias, as well as from underutilizing valuable insights due to excessive distrust. 27

In addition, by making model decisions interpretable, it becomes easier to identify systematic errors or unfair patterns that may arise from biased training data or insufficient model design. Recognizing these issues early allows for targeted mitigation strategies such as retraining the model on more diverse datasets, adjusting input variables, or applying fairness constraints. Over time, this iterative process contributes to the continuous improvement of model fairness, robustness, and clinical reliability.

XAI techniques can be broadly categorized based on their intrinsic interpretability, where the model's structure inherently provides transparency, and post hoc explainability, where surrogate techniques are used to interpret model predictions. 28 29 Table 1 shows an overview of various XAI methods.

**Table 1:** Overview of explainable AI (XAI) techniques and their relevance in pediatric surgery

XAI technique	Category	Typical output	Pediatric surgery use case examples	Strengths	Limitations
Explainability of tree-based algorithms	Intrinsic, model-specific	(Aggregated) decision paths, feature importance	Showing which features are important in sepsis prediction	Intuitive	Less accurate with complex, nonlinear data, prone to overfitting
Explainability of linear support vector machine algorithms	Intrinsic, model-specific	Decision boundaries, support vectors, feature importance	Showing which features contribute to the probability of complications after surgery	Handles high-dimensional data	Less intuitive than trees, limited for nonlinear relationships
SHAP	Post hoc, model-agnostic	Feature importance	Explaining why a model predicts high mortality risk	Consistent, both globally and locally applicable	Computationally heavy, correlation ≠ causation
LIME	Post hoc, model-agnostic	Feature importance	Explaining individual predictions of surgical risk models	Intuitive for local explanations	Stability varies, only local scope (individual explanations)
Saliency maps/Grad-CAM	Post hoc, model-specific (CNNs)	Highlighted input regions (heatmaps)	Identifying regions in brain tumor MRI classification	Intuitive visualization of what the model focuses on	Can be noisy, prone to highlighting irrelevant features
Attention mechanisms	Intrinsic (sequential models)	Highlights important time points or features	Vital sign monitoring for NEC prediction	Captures temporal patterns	Less interpretable for clinicians

### Intrinsic Interpretability

Several machine learning algorithms, such as decision trees and support vector machines (SVMs), possess inherent interpretability. These models are transparent by design, allowing clinicians to understand the decision-making process directly from the model's structure. For example, decision trees provide clear and intuitive decision paths that reflect clinical reasoning, while SVMs offer insights into the margins that separate different classes, helping to clarify which features drive the classification. In addition, both types of algorithms provide valuable information on feature importance, which is a quantification of the contribution of each input variable to the model's decision-making process. These techniques are particularly well-suited for tabular data, including patient demographics, laboratory results, and other structured information. Their ability to handle such data effectively, along with their intrinsic transparency, makes them valuable tools for supporting clinical decisions in pediatric surgery. For example, one study applied a machine learning algorithm called XGBoost to identify key predictors of rehabilitation outcomes following spinal deformity surgery in pediatric patients. 30 XGBoost is a powerful ensemble learning method based on decision trees. By aggregating the decision processes across these trees, XGBoost calculates feature importance based on how often and how strongly each feature contributes to splitting decisions throughout the ensemble. In this study, sagittal spinal parameters and patient self-image scores emerged as the most influential predictors, providing valuable insights that enhance clinical understanding and support informed decision-making.

### Post Hoc Explainability

More complex algorithms, particularly deep learning models, are often more opaque, and therefore, benefit from post hoc explainability techniques. These methods aim to elucidate the decision-making process of models that are otherwise considered black boxes. Many of these methods are model-agnostic, meaning they can be applied to any type of predictive model regardless of its internal architecture. 28

One of the most widely used post hoc explainability techniques is Shapley additive explanations (SHAP). SHAP values are grounded in cooperative game theory and provide consistent, theoretically justified importance scores for each feature, reflecting its contribution to each individual prediction. These contributions can then be aggregated to understand feature importance across the entire dataset. For example, in models predicting malnutrition risk in pediatric patients based on clinical data, SHAP identified which clinical features most strongly influence the model's risk estimates, thereby facilitating targeted interventions. 31

Local interpretable model-agnostic explanations (LIME), in contrast, explain individual predictions by fitting simple, interpretable surrogate models locally around a specific data point, approximating the complex model's behavior in that neighborhood. 32 In a study on pediatric autism diagnosis, a combination of SHAP and LIME was used to provide both global and local prediction explanations, tailored to support nuanced clinical decision-making. 33

Partial dependence plots (PDPs) are another valuable tool that illustrate the relationship between a single feature and the predicted outcome while holding all other features constant. 32 PDPs help clarify the marginal effect of that feature on model predictions and can reveal nonlinear or threshold effects important in clinical interpretation.

When dealing with medical imaging data, specialized post hoc techniques enable spatially precise explanations. 34 Saliency maps highlight image regions most influential to the model's output by computing the gradients of the output with respect to the input pixels. These sensitivity maps show how small pixel changes affect predictions, helping clinicians identify relevant anatomical areas. Gradient-weighted class activation mapping (Grad-CAM) extends this approach for convolutional neural networks by generating localization heatmaps based on gradients flowing into the last convolutional layer. 28 Grad-CAM produces more interpretable and anatomically meaningful visual explanations, facilitating clinician validation or critique of AI reasoning. For instance, in pediatric brain tumor classification using deep learning on MR images, Grad-CAM helped reveal which brain regions were critical for model decisions. 35

In sequential or longitudinal pediatric data, such as continuous vital sign monitoring or repeated clinical assessments, attention mechanisms can serve as a form of explainability by highlighting which time points or features the model focuses on most when making predictions. 36 Temporal masking techniques can systematically assess the importance of different segments of the input sequence, further elucidating the model's temporal decision patterns.

## Ethical Frameworks

Building on the understanding of XAI techniques and the critical role of identifying and mitigating bias, it becomes essential to ground its development and deployment within robust ethical frameworks. These frameworks provide guiding principles and structured approaches to ensure that XAI applications in healthcare are not only effective and transparent but also fair, responsible, and aligned with broader societal values.

Central to these ethical frameworks are the well-established Principles of Biomedical Ethics by Beauchamp and Childress, which have long guided clinical practice. 37 The four foundational pillars offer a valuable lens through which to evaluate XAI applications. 38

The first pillar, “autonomy,” refers to the respect for patients' rights to make informed decisions. Opaque AI systems reduce transparency, making it difficult for patients or parents to fully understand and consent to their treatment recommendations. This may lead to a form of AI-paternalism on top of preexisting medical paternalism, where the healthcare provider makes decisions with limited patient input while over-relying on the AI output. 38 XAI, on the other hand, allows all end-users to comprehend how its recommendations are made. This transparency, therefore, is a first step toward making informed decisions that can help preserve patient autonomy.

The second pillar, “beneficence,” means acting in the patient's best interest to promote well-being. XAI supports this by helping clinicians understand and critically evaluate AI recommendations, leading to more informed decisions that can improve outcomes in pediatric care. Similarly, “nonmaleficence,” the principle of “do no harm,” underlines the need to minimize risks and avoid unintended consequences. Explainable models help to identify errors and biases, reducing the risk of harm to vulnerable pediatric populations. However, overly simplistic or misleading explanations may foster false reassurance, potentially increasing the risk of harm. 38 Therefore, it is essential that explanations remain accurate and appropriately nuanced to truly support ethical clinical decision-making in pediatric care. Integrating fairness metrics into model evaluation can help detect and prevent systematic biases that may disproportionately harm specific patient groups.

The last pillar, “justice,” focuses on fairness and equal access to benefits and risks. XAI increases transparency and helps uncover biases that may disadvantage certain groups, promoting equitable care. Nonetheless, if explanations are poorly designed or too complex, or if healthcare professionals show an overreliance on XAI, they may obscure inequities or hinder the detection of unfair treatment, thereby undermining the principle of justice. 38

Taken together, these principles highlight the essential ethical requirement for explainability in AI systems, especially in pediatric care, where patients are unable to advocate for themselves and depend on caregivers and clinicians to make fully informed decisions on their behalf. Building on these ethical imperatives, a range of international frameworks and legal regulations have translated these principles into concrete requirements for transparency, accountability, and responsible use of AI.

In 2019, the European Commission's High-Level Expert Group on AI published the EU Ethics Guidelines for Trustworthy AI. 39 These guidelines define seven key requirements for trustworthy AI systems: human agency and oversight, technical robustness and safety, privacy and data governance, transparency, diversity and fairness, societal well-being, and accountability. Explainability plays a vital role in several of these domains by allowing users to understand, interpret, and critically evaluate AI outputs.

Similarly, the World Health Organization's Guidance on Ethics and Governance of AI for Health emphasizes six core principles: safety, effectiveness, equity, transparency, accountability, and responsiveness. 40 XAI contributes directly to these principles by improving interpretability, reducing the risks associated with opaque or biased decision-making, and ensuring that AI systems can be integrated responsibly into clinical workflows.

Crucially, the ethical expectations around explainability have now been enshrined in law through the European Union AI Act, which came into force in August 2024. 41 This legislation classifies medical AI systems as “high-risk,” imposing strict legal requirements for transparency, human oversight, and explainability. The Act mandates that AI developers and healthcare providers must ensure that AI outputs are interpretable and that decisions supported by AI can be clearly justified. This legal framework effectively makes XAI a compulsory standard in clinical practice, aiming to protect patient safety, uphold trust, and provide clear accountability pathways in healthcare AI deployment.

In addition to these foundational frameworks, the ACCEPT-AI framework has emerged as a crucial tool in guiding the responsible deployment of AI in pediatrics. 42 This framework addresses the practical challenges of integrating AI systems in clinical care for children by promoting continuous human oversight and fostering trust through clear, age-appropriate explanations. This framework thus complements existing ethical guidelines by offering an operationalized approach to embedding explainability and ethical safeguards tailored to pediatric contexts into everyday AI use.

Together, these ethical principles, international guidelines, and binding legal requirements underscore that explainability is not only a desirable feature but a fundamental obligation, especially critical in pediatric care, where vulnerable patients depend entirely on responsible, transparent decision-making.

## Necessity of Benchmarks

Despite its clear benefits and the essential role it plays within ethical frameworks, XAI faces several inherent challenges that complicate its implementation and effectiveness. One major limitation is that XAI methods typically highlight which features contribute to a model's prediction but do not necessarily establish causal relationships. 14 This can be misleading if clinical interventions are based solely on correlational insights, potentially resulting in ineffective or inappropriate treatments. Confirmation bias poses a significant concern in this regard, as practitioners often assume that the explanations provided by the AI system correspond to the reasons they expect or desire to see. 43

Another challenge lies in translating technical explanations into language that is accessible and meaningful not only to clinicians, but also parents and patients. 14 44 Overly technical or jargon-heavy explanations risk distancing users, reducing trust and acceptance. Therefore, explanations must balance completeness with context-appropriate conciseness. For these reasons, the integration of XAI models into pediatric surgery demands rigorous validation frameworks to ensure their explanations are both reliable and clinically meaningful. Benchmarks serve as critical tools in this context, providing standardized metrics and datasets to systematically evaluate and compare the performance and interpretability of various XAI approaches. Such benchmarks typically include curated datasets, well-defined tasks, and quantitative metrics designed to assess key dimensions of explainability, including interpretability and fidelity. 28 32

Iteratively using benchmarks to identify which explainability techniques are most effective in specific clinical scenarios supports the continuous refinement of XAI methods. Importantly, the development and use of rigorous benchmarks closely align with international ethical frameworks and regulatory requirements. By ensuring that explainability methods meet these ethical standards, benchmarks contribute not only to scientific rigor but also to the responsible and lawful implementation of AI.

Examples of benchmarks for XAI are “BenchXAI” and “XAIB.” 45 46 BenchXAI focuses on multi-modal biomedical data, including medical imaging, genomics, and clinical records. XAIB, in contrast, provides a general-purpose, modular framework to evaluate post hoc explainability techniques across various domains. Both systematically compare popular XAI methods and offer metrics to evaluate explanation quality. By quantifying explanation consistency and relevance, these benchmarks help to identify which techniques are most appropriate for tasks such as disease classification, lesion localization, or treatment recommendations.

However, existing benchmarks do not address the unique challenges inherent to pediatrics. Establishing such a benchmark is crucial for several reasons. Pediatric data is inherently complex due to rapid developmental changes, age-specific disease manifestations, and typically smaller, more heterogeneous cohorts. 14 Furthermore, AI-generated explanations in pediatrics must be understandable not only to clinicians but also to parents and, in some cases, to children themselves. This demands explanations that are both medically sound and accessible to nonexpert audiences. In addition, trust and transparency are particularly critical in pediatric care, where decisions often involve parents making informed choices on behalf of their children. A dedicated pediatric XAI benchmark would enable systematic evaluation and comparison of methods in terms of clinical relevance, robustness, and usability, ultimately facilitating safer and more ethical integration of AI in pediatric surgery.

## Future Perspectives and Recommendations

A recent systematic review on AI applications in pediatrics highlighted that explainability remains significantly underdeveloped. 2 Only a minority of studies incorporated XAI methods, and most lacked formal benchmarking or standardized tasks to evaluate explainability rigorously. Similarly, another study found that only 44% of AI models in pediatric surgery were interpretable, and only 6% were both interpretable and externally validated. 4 This illustrates a critical gap in the adoption and validation of XAI within pediatrics.

Consequently, there is an urgent need to further develop and validate intrinsically interpretable models as well as causal inference methods to provide robust and clinically meaningful explanations. 28 Similarly, explainability efforts in pediatric AI should be advanced through the development of dedicated benchmarks and standardized evaluation protocols that address the unique complexities of pediatric data and clinical settings.

Furthermore, existing benchmarks predominantly rely on retrospective or simulated datasets, which do not fully capture the intricacies of real-world clinical workflows. Effective evaluation of explainability must therefore extend to prospective human-in-the-loop studies that actively engage end-users such as pediatricians and caregivers. Additionally, it is imperative to assess the impact of explainability on long-term clinical outcomes through prospective studies. 28 By maintaining a focus on practical applicability and end-user engagement, XAI can better fulfill ethical and regulatory requirements while contributing substantively to the advancement of pediatric surgery.

## Conclusion

XAI offers significant potential to enhance transparency and trust in pediatric healthcare by enabling more interpretable and accountable AI-driven decisions. Effectively addressing biases and thoroughly evaluating intrinsic and post hoc explainability approaches are crucial to developing fair and context-appropriate models for pediatric use. Ethical frameworks, including international guidelines and legal regulations, provide essential principles to guide the responsible implementation of AI in this sensitive domain. Furthermore, the establishment of standardized benchmarks is indispensable for objectively assessing AI performance and explainability. Moving forward, collaborative efforts across disciplines and active involvement of end-users will be key to advancing XAI solutions that are both technically robust and ethically sound, ultimately improving outcomes for pediatric patients.
